# Resolving Toxic DNA repair intermediates in every *E. coli* replication cycle: critical roles for RecG, Uup and RadD

**DOI:** 10.1093/nar/gkaa579

**Published:** 2020-07-09

**Authors:** Zachary J Romero, Stefanie H Chen, Thomas Armstrong, Elizabeth A Wood, Antoine van Oijen, Andrew Robinson, Michael M Cox

**Affiliations:** Department of Biochemistry, University of Wisconsin-Madison, Madison, WI 53706, USA; Biotechnology Program, North Carolina State University, Raleigh, NC 27695, USA; Molecular Horizons Institute and School of Chemistry, University of Wollongong, Wollongong, Australia; Illawarra Health and Medical Research Institute, Wollongong, Australia; Department of Biochemistry, University of Wisconsin-Madison, Madison, WI 53706, USA; Molecular Horizons Institute and School of Chemistry, University of Wollongong, Wollongong, Australia; Illawarra Health and Medical Research Institute, Wollongong, Australia; Molecular Horizons Institute and School of Chemistry, University of Wollongong, Wollongong, Australia; Illawarra Health and Medical Research Institute, Wollongong, Australia; Department of Biochemistry, University of Wisconsin-Madison, Madison, WI 53706, USA

## Abstract

DNA lesions or other barriers frequently compromise replisome progress. The SF2 helicase RecG is a key enzyme in the processing of postreplication gaps or regressed forks in *Escherichia coli*. A deletion of the *recG* gene renders cells highly sensitive to a range of DNA damaging agents. Here, we demonstrate that RecG function is at least partially complemented by another SF2 helicase, RadD. A *ΔrecGΔradD* double mutant exhibits an almost complete growth defect, even in the absence of stress. Suppressors appear quickly, primarily mutations that compromise *priA* helicase function or *recA* promoter mutations that reduce *recA* expression. Deletions of *uup* (encoding the UvrA-like ABC system Uup), *recO*, or *recF* also suppress the *ΔrecGΔradD* growth phenotype. RadD and RecG appear to avoid toxic situations in DNA metabolism, either resolving or preventing the appearance of DNA repair intermediates produced by RecA or RecA-independent template switching at stalled forks or postreplication gaps. Barriers to replisome progress that require intervention by RadD or RecG occur in virtually every replication cycle. The results highlight the importance of the RadD protein for general chromosome maintenance and repair. They also implicate Uup as a new modulator of RecG function.

## INTRODUCTION

The replication of genomic DNA is an essential process that is carried out by a highly complex and regulated assembly of proteins called the replisome. As replication proceeds, the replisome encounters impediments. Exogenous damage from the environment, protein–DNA complexes, reactive oxygen species (ROS), and genotoxic agents can cause replisome stalling and fork collapse. If improperly repaired, lesions and breaks can produce mutagenesis. Mutagenesis in turn can give rise to human disease. In any organism, replication rarely, if ever, completes uninterrupted (1–3). The potential biological consequences and frequency of replication conflicts underscores the importance of understanding DNA repair and replication enzymes.

**Figure 1.**
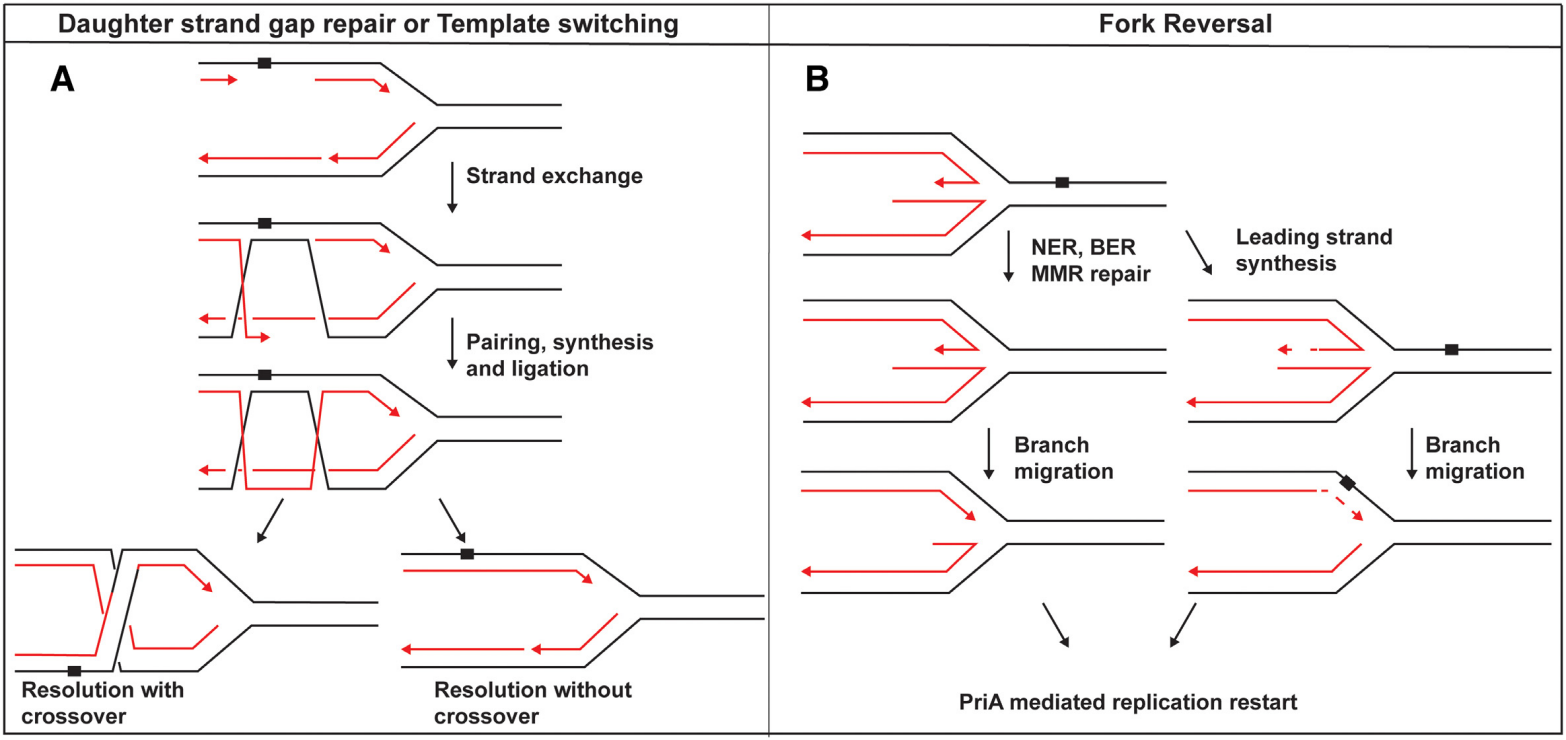
Possible fates of abandoned replication forks. (**A**) The formation of post replication gaps by lesion skipping. Gaps generated by lesion skipping can be filled either by RecA-mediated homologous recombination or RecA-independent template switching. Synthesis is initiated from an undamaged template. Resolution of this intermediate can yield either crossover or non-crossover products. (**B**) The process of replication fork reversal and Holliday junction formation. The lesion can be re-incorporated into the parental duplex to grant other repair pathway access. The lesion can also be bypassed by nascent strand template switching on the free end of the Holliday junction.

In most bacteria, replication initiates from a single origin called *oriC*. From there the two replication forks move bi-directionally on the circular chromosome until meeting at the terminus opposite of the origin. When a lesion is encountered by a replisome, repair can take many forms (Figure 1). Polymerase switching is a well-documented process in vitro that allows for lesion bypass by translesion DNA synthesis (4,5). In contrast, some lesions can be passed over by lesion skipping on either the leading or lagging strand. This consists of re-priming the replicative polymerase downstream of a roadblock for continued DNA synthesis (6–8), leaving the lesion behind in a gap. The postreplication gap left behind is filled by RecA in RecFOR mediated gap repair or RecA-independent template switching (9,10).

If the replisome is unable to bypass a lesion it can disassociate, leaving behind an abandoned fork. Repair enzymes can then access the fork and re-anneal the parental duplex creating a Holliday Junction (HJ) in a process known as fork reversal or fork regression. Fork reversal is a frequent process, occurring in 25–40% of cells treated with a Topoisomerase I inhibitor (11). An important feature of fork reversal is the re-incorporation of the lesion back into the parental duplex. This allows Mismatch Repair (MMR), Base Excision Repair (BER), or Nucleotide Excision Repair (NER) enzymes to remove any lesion in question (Figure 1B). As an alternative, synthesis can occur on the free end of the reversed junction if the lagging strand has been replicated further than the leading strand. Both processes require branch migration back to a suitable fork substrate for PriA-mediated restart. Exonucleases may also digest the protruding arm on junctions to restore a fork (12). However, this would negate any template switch synthesis that has occurred. In bacteria, RecG, RecQ, RuvAB and RecA are all capable of or implicated in reversing replication forks (13–17). In humans, SMARCAL1, HLTF, RAD51 and ZRANB3 are enzymes involved in fork reversal and branch migration (10,11,18).

In *Escherichia coli*, the SF2 helicase RecG has emerged as a key player in this process (19–25). *In vitro*, RecG can reverse forks and, alternatively, branch migrate the resulting Holliday junction back to a fork structure. *In vivo*, a *recG* null strain is still capable of fork reversal, suggesting little or no involvement of RecG in the initial fork processing. However, the products of fork reversal, Holliday junctions, accumulate at sites of replisome stalling (17). This suggests that an important role of RecG in fork repair is to remodel Holliday junctions back to fork structures after repair.

Cells lacking *recG* function grow normally but are sensitive to many DNA damaging agents. When a *ΔrecG* strain is treated with UV, suppressor mutations arise in *priA* that may function by altering the PriA helicase activity without compromising the capacity of PriA to load DnaB for replication restart (12,26–31). The toxicity of a fully helicase-competent PriA in UV treated *ΔrecG* cells has yet to be fully explained. *In vitro*, some suppressor mutations render PriA incapable of unwinding the nascent lagging strand at a fork without a leading strand present. The emerging model is that, after repair, RecG restores a replication fork that has a nascent leading strand end in proximity to the junction. An end thus positioned correctly orients PriA to facilitate DnaB helicase loading and replication restart (3,15,19,26,28). Without a leading strand, PriA can incorrectly unwind the parental duplex. The presence of the single stranded DNA binding protein, SSB, allows for bypass of this leading strand requirement (30). High concentrations of SSB and its presumed presence at an abandoned replication fork suggest that toxicity is not from parental duplex unwinding (32).

We have identified an apparently complementary relationship between another SF helicase, RadD and RecG. RadD shares significant homology to the *E. coli* SF2 helicases RecQ and RecG. Previous work has shown RadD suppresses crossovers that can occur in postreplication gaps and can bind forked DNA structures (33). It is important for survival during tobramycin and ionizing radiation treatment. RadD also has a functional interaction with the C-terminal tail of the SSB (34–36), as does RecG and RecQ (37,38). This interaction places the primary function of RadD at the fork or single strand gaps. We also identified several suppressors of this phenotype that have given us more insight into the functions of RadD, PriA, Uup, RecA and RecG.

## MATERIALS AND METHODS

### Strain construction

Strains used in this report are in Table 1. A modification of the method by Datsenko & Wanner (39) was used to construct chromosomal gene knockouts and point mutations. The plasmid pEAW507 contains a kanamycin (Kan) cassette flanked by FRT recognition sites for the FLP recombinase (pJFS42 mutant FRT-Kan^R^-wt FRT) was the template for gene deletions. PCR amplification across this region was carried out using primers with (a) 21 nucleotides of sequence complementary to one end of the cassette, and (b) an additional 50 nucleotide complementary sequence to regions flanking the gene of interest. Gel-purified PCR product was electroporated into cells containing pDK46, which expresses the lambda red recombinase. Recombinase expression was induced by the addition of l-arabinose. Kanamycin resistant colonies were screened for ampicillin sensitivity and used as a template for colony PCR confirmation. The Kan^R^ cassette was removed by transforming strains with a plasmid that harbors the FLP recombinase (pLH29). For strains containing multiple deletions, P1 transduction was used to introduce multiple alleles. The process of P1 transduction consisted of plating the initial transductants on LB + antibiotic. Resulting transductans were then streaked again on LB + antibiotic to ensure resistance. All strain constructions were confirmed by PCR amplification across all relevant deletion sites and/or direct sequencing.

**Table 1.** Strains used in this study

Strain	Genotype	Parent strain	Source/technique
MG1655	*uup^+^ radD^+^ recG^+^*	-	(65,66)
EAW9	ΔrecA recG-	MG1655	△recA to recG- 133FRT#1 from pEAW324 template
EAW114	ΔrecO	MG1655	Lambda RED recombination
EAW232	*Founder* Δ*e14*Δ*radD*	MG1655	Lambda RED recombination
EAW242	*Founder* Δ*e14*Δ*uup*	MG1655	Lamda RED recombination
EAW368	founderΔe14 Δ*radD* recG-	MG1655	EAW232 transduced to recG- with P1 grown on EAW9 Kan^R^
EAW401	founderΔe14 ΔruvB	MG1655	Lambda RED recombination
EAW408	△*lacIYZA*	MG1655	Lambda RED recombination
EAW505	Δ*recG*	MG1655	Lambda RED recombination
EAW526	Δ*radD*	MG1655	Transduction of MG1655 with P1 grown on EAW232 Kan^R^
EAW531	Δ*recG*Δ*radD*	MG1655	Transduction of EAW505 with P1 grown on EAW526 Kan^R^
EAW552	*founder*Δ*e14* Δ*radD recG-, priA S278A-Tet*	MG1655	EAW368 suppressor#1 with wtFRT-TetR-wt FRT after priA
EAW553	*founderΔe14* ΔradD *recG-, priA A520P-Tet*	MG1655	EAW368 suppressor#5 with wtFRT-TetR-wt FRT after priA
EAW629	Δ*recF*	MG1655	Lambda RED recombination
EAW1073	P_recA_ A → G	MG1655	Lambda RED recombination
EAW1075	Δ*radD P_recA_ A → G*	MG1655	Transduction of EAW526 with P1 grown on EAW1073 Kan^R^
EAW 1087	Δ*radD P_recA_ A → G* Δ*recG*	MG1655	Transduction of EAW1075 with P1 grown on EAW505 Kan^R^
EAW1097	MG1655 Δ*ruvB*	MG1655	Transduction of MG1655 with P1 grown on EAW401 Kan^R^
EAW1100	Δ*radD* Δ*lac IZYA*	EAW526	Transduction of EAW526 with P1 grown on EAW408 Kan^R^
EAW1102	*MG1655* Δ*recG*Δ*lac IZYA*	MG1655	Transduction of EAW505 with P1 grown on EAW408 Kan^R^
EAW1104	*MG1655* Δ*radD*Δ*uup*Δ*lac IZYA*	MG1655	Transduction of ZJR04 with P1 from EAW408 Kan^R^
EAW1132	Δ*radD*Δ*lac IZYA*Δ*uup*	EAW1100	Transduction of EAW1100 with P1 grown on EAW242 Kan^R^
ZJR01	Δ*uup*°	MG1655	Transduction of MG1655 with P1 grown on EAW242 Kan^R^
ZJR04	Δ*uup*Δ*radD*	MG1655	Transduction of ZJR01 with P1 grown on EAW232 Kan^R^
ZJR10	Δ*uup*Δ*recG*	MG1655	Transduction of ZJR01 with P1 grown on EAW505 Kan^R^
ZJR17	Δ*uup*Δ*recG*Δ*radD*	MG1655	Transduction of ZJR10 with P1 grown on EAW 232 Kan^R^
ZJR20	Δ*uup*Δ*ruvB*	MG1655	Transduction of ZJR01 with P1 grown on EAW 401 Kan^R^
ZJR 22	Δ*radD*Δ*LacIZYA P_recA_ A → G*	MG1655	Transduction of EAW1100 with P1 from EAW 1073 Kan^R^
ZJR29	Δ*radD priA S278A*	MG1655	Transduction of EAW526 with P1 grown on EAW552 Tet^R^
ZJR31	Δ*radD*Δ*lacIZYA priA S278A*	MG1655	Transduction of EAW 1100 with P1 grown on EAW552 Tet^R^
ZJR32	Δ*radD*Δ*lacIZYA priA A520P*	MG1655	Transduction of EAW 1100 with P1 grown on EAW553 TetR^R^
ZJR34	Δ*recG priAS278A*	MG1655	Transduction of EAW505 with P1 grown on EAW522 Tet^R^
ZJR35	Δ*radD* priA S278A △*recG*	MG1655	Transduction of ZJR29 with P1 grown on EAW505 Kan^R^
ZJR36	Δ*radD*Δ*lacIZYA priA* S2*78A* Δ*recG*	MG1655	Transduction of ZJR31 with P1 grown on EAW 505 Kan^R^
ZJR37	Δ*radD*Δ*lacIZYA priA A520P* Δ*recG*	MG1655	Transduction of ZJR32 with P1 grown on EAW505 Kan^R^
ZJR40	Δ*radD*Δ*LacIZYA P_recA_ A → G* Δ*recG*	MG1655	Lambda RED recombination
ZJR41	ΔrecG priA A520P	MG1655	Transduction of EAW505 with P1 grown on EAW553 Tet^R^
ZJR42	Δ*radD priA A520P* Δ*recG*	MG1655	Transduction of ZJR41 with P1 grown on EAW232 Kan^R^
ZJR49	Δ*radD*Δ*lacIYZA*Δ*recO*	MG1655	Transduction of EAW1100 with P1 grown on EAW114 Kan^R^
ZJR50	Δ*radD*Δ*recG*Δ*lacIYZA*Δ*recO*	MG1655	Transduction of ZJR49 with P1 grown on EAW505 Kan^R^
ZJR51	△*radD*△*recF*△*lacIYZA*	MG1655	Transduction of EAW1100 with EAW629 Kan^R^
ZJR52	Δ*radD*Δ*recF*Δ*lacIYZA*Δ*recG*	MG1655	Transduction of ZJR51 with P1 from EAW505 Kan^R^
ZJR54	Δ*uup*Δ*rnhA*	MG1655	Transduction of ZJR01 with P1 grown on JW0204 from Keio collection (67) Kan^R^
ZJR55	*dnaA(46)ts*	MG1655	Transduction of MG1655 with P1 grown on MG1655 dnaA(46)ts (68)
ZJR56	Δ*uup* dnaA(46)ts		Transduction of ZJR01 with P1 grown on ZJR55
ZJR57	dnaA(46)ts + Δ*rnhA*		Transduction of ZJR55 with P1 grown on JW0204
ZJR58	Δ*uup*Δ*rnhA dnaA(46)ts*	MG1655	Transduction of ZJR54 with P1 grown on ZJR55 (68).

To construct the Δ*radD*Δ*recG* strain, the *radD* deletion was introduced into the *recG* deletion strain by P1 transduction. Very small colonies appeared after growth overnight on LB plates containing Kanamycin. Multiple cultures from transductant colonies were grown overnight. Turbidity at this point was minimal but detectable. Several minimally turbid cultures were spun down, resuspended in 1 ml LB, and frozen. The presence of the two deletions was confirmed both by PCR amplification and by direct sequencing of the PCR product. This was the stock used for all experiments. Unless stated otherwise, all subsequent growth curves and spot plates were initiated by inoculating a fresh tube of media from the same frozen aliquot of Δ*radD*Δ*recG* cells.

### Growth curves and SOS induction assays

In order to minimize growth before testing all growth curves had to be initiated from freezer stocks. 3 ml of LB was inoculated to a minimum OD_600_ of 0.01. Each culture was then diluted to give a starting OD_600_ of 0.005 and 100 μl of each culture was added to a 96-well plate. Growth was monitored at 37°C while shaking in a H1 Synergy Biotek plate reader. Optical density readings were taken every 10 min for 24 h.

For SOS induction assays, we utilized a plasmid containing SuperGlo GFP under control of the *recN* promoter (pEAW903). Each strain was transformed with pEAW903 and cultures were diluted to an initial OD_600_ of 0.005. SOS induction was monitored by measuring GFP fluorescence every 10 min for 24 h along with OD_600_ readings at 37°C while shaking in a H1 Synergy Biotek plate reader. Data was exported and data graphed using GraphPad Prism Software. Statistical analysis was based on at least three replicates in all experiments.

### Mini-F pRC-7 plasmid assay

The pRC7 plasmid is a lac^+^ mini-F low copy derivative of pFZY1 (40). pJJ100 that harbors *recG* was a generous gift from Christian Rudolph and constructed as described previously (41). All strains were transformed with pJJ100 before adding the final mutation to be tested. For example, a Δ*radD* strain was transformed with pJJ100 and plated on 0.5× (Amp50) ampicillin. With plasmid selection present *recG* was deleted using P1 transduction and by plating on Kan 40 and Amp 50. This ensured that *recG* was always present and removed the chance of suppressor mutations arising. Once constructed, 3 ml overnights of each strain with the pJJ100 plasmid were set and allowed to grow overnight for 16 h. The following day 5 ml fresh LB was inoculated with 50 μl overnight; at this point antibiotic was withheld. Cultures were grown to an OD_600_ of 0.2 and placed on ice for a minimum of 5 min, serially diluted in 1× PBS Buffer (137 mM NaCl, 2.7 mM KCl, 10 mM Na_2_HPO_4_, 1.8 mM KH_2_PO_4,_ 1 mM CaCl_2_ and 0.5 mM MgCl_2_) and spread on X-gal IPTG plates. Plates were allowed to grow for a strict 16 h for initial blue and white colony counting. Plates were then allowed to grow for an addition 8 hours and colonies were recounted. All experiments were repeated at least three times with comparable results.

### Sensitivity assays

All strains were grown in 3 ml LB culture overnight at 37°C while shaking. The following day 50 μl of overnight was used to inoculate 5 ml LB and grown to an OD_600_ of 0.2 while shaking at 37°C. Cultures were serially diluted in 10× steps to 10^−6^ in 1× PBS buffer in a 96-well plate. LB agar plates were made the day of the assay and kept in dark to prevent break down of DNA damaging agents. A total of 10 μl of each dilution was plated for all strains and the plates were photographed after growth at 37°C overnight. All experiments were repeated at least three times with comparable results.

### Bright-field microscopy

For all measurements of cell filamentation, wide-field microscopy was conducted on an inverted microscope (IX-81, Olympus with a 1.49 NA 100× objective). Bright-field images were collected on a 512 × 512 pixel EM-CCD camera (C9100-13, Hamamatsu). For imaging of all strains we used glass coverslips functionalized with 3-amino-propyl-triethoxysilane (APTES, Alfa Aeser) to immobilize cells on the coverslip surface.

Coverslips were first sonicated for 30 min in 5M KOH to clean and activate the surface of the coverslip. Coverslips were then rinsed thoroughly with MilliQ water, then treated with 1 ml 5% (v/v) of APTES in MilliQ water for 10 min. Subsequently, coverslips were rinsed with ethanol twice and sonicated in ethanol for a further 20 s. Finally, functionalized coverslips were rinsed with MilliQ water and dried in a jet of N_2_ and stored under vacuum prior to use.

### Live-cell imaging

For all imaging experiments, cells were grown overnight at 37°C with shaking in EZ rich defined medium (Teknova) that contained 0.2% (w/v) glucose. Overnight, saturated cultures were reset 1 in 1000 μl EZ glucose and grown out for 3 h before imaging. To initiate imaging, 20 μl of cells were loaded onto an APTES functionalized coverslip, sandwiched with a KOH cleaned coverslip and allowed to associate with the surface before being imaged. A single bright-field image (34 ms exposure) was taken at multiple fields of view to determine cell lengths and filamentation.

### Analysis of cell filamentation

Bright-field images of all strains were imported into MicrobeTracker 0.937 (42), a MATLAB script, was used to create cell outlines as regions of interest (ROI’s). Cell outlines were manually created and designated via MicrobeTracker to ensure accuracy and that only non-overlapping, in-focus cells were selected for analysis. ROI’s were then exported Microsoft Excel to define cell parameters including cell length.

## RESULTS

### Cells lacking both RadD and RecG exhibit a severe growth defect

A *radD* deletion does not confer a significant growth defect on the host cell. In order to gain insight into the role of RadD *in vivo*, we have begun to explore its relationship with other cellular DNA helicases. We previously showed that removing both RadD and RadA function did not affect growth under standard conditions. However, when treated with Ciprofloxacin or UV, the *ΔradAΔradD* strain exhibited a significant loss in viability (34). Cells lacking both RadD and RecG function exhibited a more serious loss of viability, but that phenotype was not extensively characterized (34). The properties of the Δ*radD*Δ*recG* strain provided the starting point for the current study.

**Figure 2.**
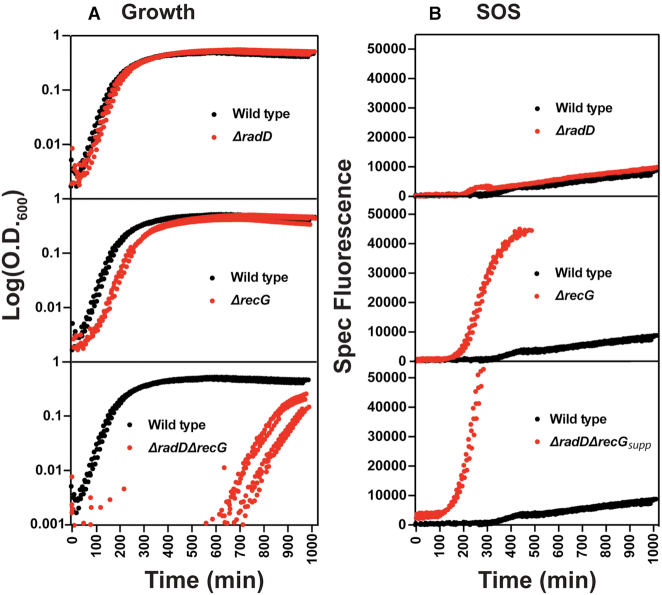
Growth curves and SOS induction of *recG* and *radD* single and double mutants. (**A**) A minimum of 3 Log scale OD_600_ versus time traces of each strain shown in comparison to a wild type control. Wild type is always shown in black with mutants appearing in red. (**B**) SOS traces of *recG* and *radD* single and double mutants over time. The y-axis is the fluorescent signal divided by the optical density of the corresponding replicate. Experimental setup is further described in methods. The Δ*radD*Δ*recG* strain is designated Δ*radD*Δ*recG*_supp_ to highlight the presence of a suppressor.

We began by measuring the growth rate of a Δ*radD*Δ*recG* strain and the related single deletion strains. All samples were normalized to an initial OD_600_ of 0.005 before being set for monitored growth to ensure that the initial number of cells per culture was comparable. Wild type, Δ*radD*, and Δ*recG* strains grow unhindered as expected (Figure 2A), although deleting *recG* produces a slight lag in growth and reduction in growth rate. Deleting both *radD* and *recG* creates an extended lag phase lasting up to 8 h or more. Once growth begins, the culture approached saturation at a rate similar to a Δ*recG* strain. Isolates from the saturated Δ*radD*Δ*recG* culture exhibited the same colony size as wild type cells when grown on plates from single cells overnight. These same isolates consistently did not exhibit the long growth lag of the original strain. This suggested the presence of suppressor mutations.

The induction of SOS in the absence of damage was also measured for each strain. To monitor SOS induction, we used a plasmid harboring an early SOS-sensitive *recN* promoter that controls GFP expression. One caveat of this assay is the reporter plasmid carriers a pMB1 origin that might affect plasmid stability. It is important to note, all the strains were grown to saturation overnight. Each strain was diluted in fresh media to give a starting OD_600_ of 0.005. This additional growth prior to measurement will cause the Δ*radD*Δ*recG* strain to accumulate suppressors and grow much faster than observed in Figure 2A. Deleting *radD* produced no increased SOS induction in the absence of stress compared to wildtype (Figure 2B). A Δ*recG* strain exhibited substantial SOS induction, again in the absence of stress. The signal halts after ∼500 min because the GFP signal saturates the capacity of the plate reader. The Δ*radD*Δ*recG* strain exhibited a higher induction of SOS before saturating our plate reader 3 h faster than a *recG* deletion alone. This signal is coming from a strain that has accumulated a suppressor, as detailed later in this study. The Δ*radD*Δ*recG* strain is thus designated Δ*radD*Δ*recG*_supp_ to highlight this status. These results support the idea that in the absence of either RadD or RecG, the requirement for the other activity is increased.

**Figure 3.**
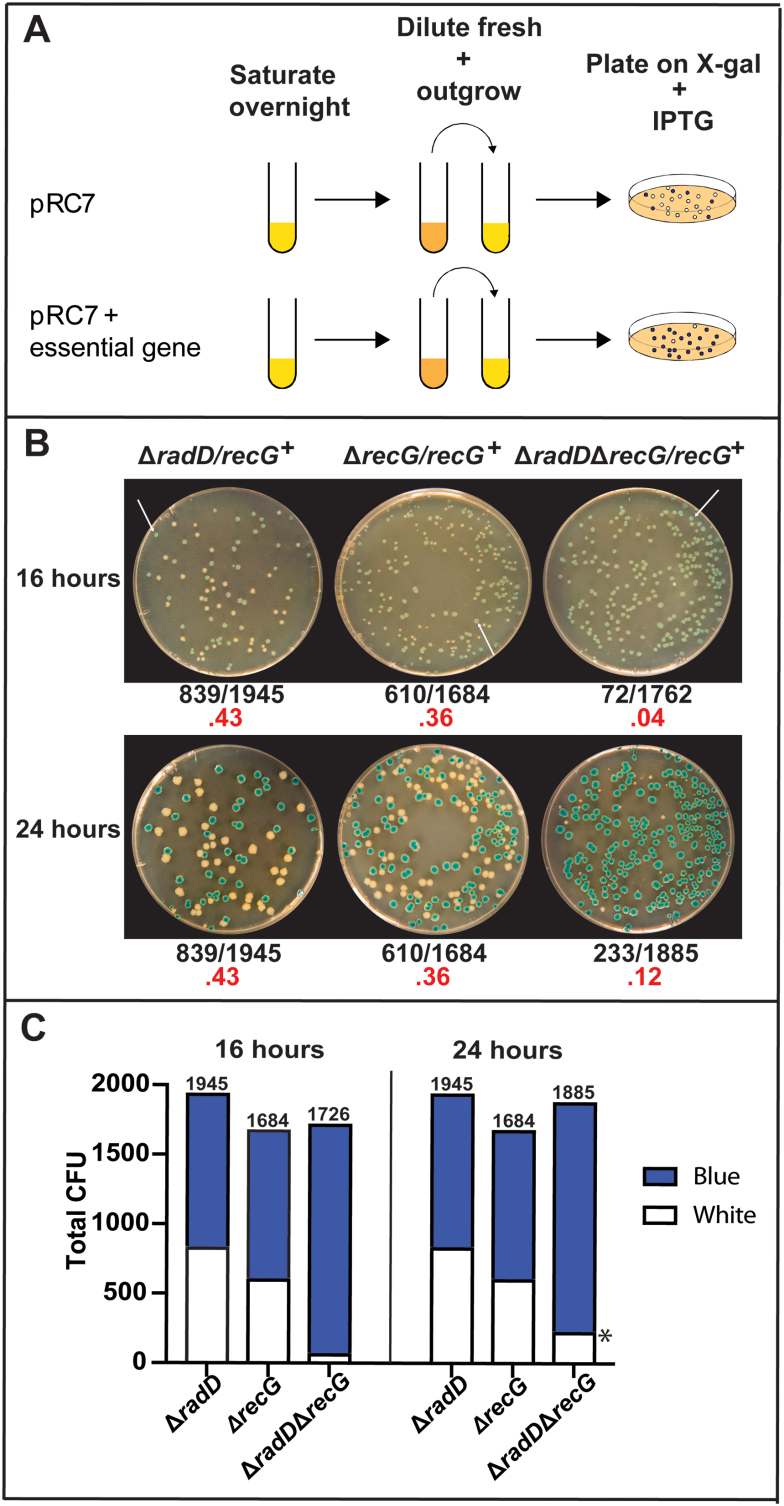
(**A**) Schematic of pRC7 synthetic lethality assay. (**B**) Images of results from the *recG* and *radD* single and double mutant pRC7 assay. Images of plates were taken at both 16 and 24 h to show the accumulation of white colonies after significant time in a *radD recG* double mutant. The white arrows at 16 h incubation point to blue colonies. Frequency of white colonies is highlighted in red underneath each image. (**C**) Stacked bar graph showing the total colony counts from each strain and their distribution of either white or blue colonies. The * denotes the appearance of suppressors that came up after 16 h colony counts.

To further characterize this genetic relationship, we utilized a pRC7 synthetic lethality assay. The pRC7 plasmid is a mini-F derivative that contains the lac operon. The unstable nature of the mini-F element allows it to be rapidly cured by growing cells in media without selection (40). Placing an essential or conditionally essential gene on the plasmid will act as a form of selection in the absence of antibiotic. The resulting colonies, when plated on X-gal and IPTG, will be blue if the cells retain the plasmid or white if they have lost it (Figure 3A). We used a previously reported pRC7 construct called pJJ100, featuring an expressed wild type copy of *recG* (41). Note that plasmid stability may be affected when deleting multiple DNA repair genes. We use this assay only to underscore the importance of retaining the RecG-encoding plasmid for growth in the Δ*radD*Δ*recG* background. We do not draw conclusions from subtle differences in the retention % of the pRC7 plasmid. Cells lacking either *radD* or *recG* alone lost the plasmid expressing RecG and produced white colonies at a frequency of 43% and 35%, respectively, of the total after 16 hours of growth (Figure 3B, C). In contrast, when the Δ*radD*Δ*recG* strain was plated, white colonies represented only 4% of the total after 16 h of growth. In addition, the white colonies were much smaller than the blue colonies, again suggesting the appearance of suppressors. To monitor suppressor appearance, we took colony counts at both 16 and 24 h. At 24 h of growth, the Δ*radD*Δ*recG* plates accumulated additional white colonies. When five of these colonies were cultured again, each reproducibly displayed a restored growth phenotype. Both the *radD* and *recG* single deletion strains produced the same colony counts at 16 and 24 h, with no evident distinction in colony size between white and blue colonies.

### Viability in Δ*radD*Δ*recG* is restored by suppressor mutations in *priA* and the *recA* promoter (*P_recA_*)

**Figure 4.**
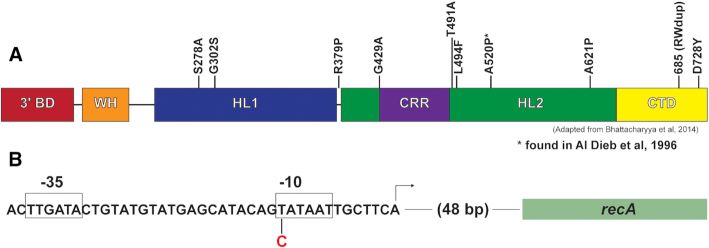
(**A**) Domain layout of PriA protein and location of the 10 suppressor mutations isolated. The layout of the PriA protein is adapted from Bhattacharyya *et al.* (**B**) Layout of the RecA promoter and position of the *P_recA_* suppressor mutation in the –10 region. Abbreviations are as follows: 3′ BD = 3′ binding domain, WH = winged helix domain, HL1 = helicase lobe 1, CRR = cysteine rich region, HL2 = helicase lobe 2, CTD = C-terminal domain.

The larger colony size in Δ*radD*Δ*recG* after the extended lag phase strongly suggested the presence of suppressor mutations. Eleven of the putative suppressor colonies were isolated. Mutations in *priA* suppress the DNA damage sensitivity of *recG* mutants (12,26–30). Based on this precedent, we first sequenced the *priA* gene in each isolate and found *priA* gene mutations in 10 of the 11 (Figure 4A). This suggested that *priA* mutations are the most common suppressors of Δ*radD*Δ*recG* (as confirmed below). Unlike the suppression observed in *recG* mutants alone, the suppression observed here occurs in the absence of elevated levels of DNA damage. We chose to do further studies on PriA S278A and PriA A520P. The latter suppressor has been observed in a previous study involving *recG* mutant suppression (31).

One of the spontaneous suppressor isolates failed to produce a *priA* mutation when that gene was sequenced. This isolate was subjected to genomic sequencing that revealed a mutation in the *recA* promoter (*P_recA_*). The base change is a T to C in the first position of the six-nucleotide Pribnow box sequence (Figure 4B). Mutations in this position will result in reduced expression of *recA* (43,44). RecA is the central recombinase in *E. coli* that facilitates homologous pairing in double strand break and daughter strand gap repair (45,46). The involvement of RecA in abandoned fork processing has been documented (13,41). Additional mutations found in the genomic sequencing of this suppressor strain are listed in Supplementary Table S1.

### Validation of spontaneous suppressor mutations

**Figure 5.**
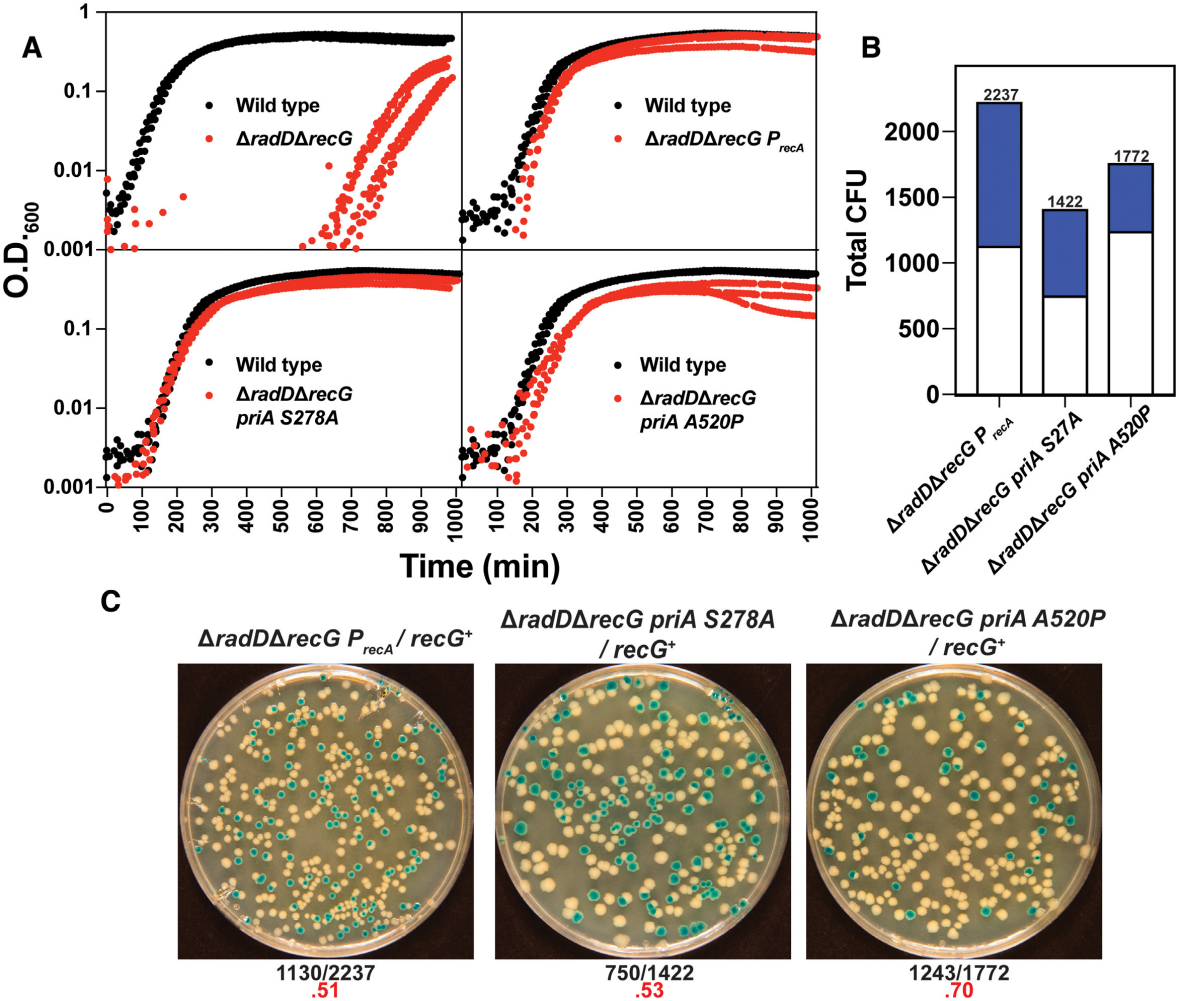
(**A**) A minimum of 3 Log scale OD_600_ versus time traces of Δ*radD*Δ*recG* strain with either *priA S278A, priA A520P or P_recA_* suppressor mutations (red) in comparison to a wild type control (black). (**B**) Stacked bar graph quantifying results of pRC7 assay of Δ*radD*Δ*recG* with *priA S278A, priA A520P or P_recA_* mutations. (**C**) Images of plates after 24 h for Δ*radD*Δ*recG* with *priA S278A, priA A520P or P_recA_* suppressor mutations to show loss of plasmid increase. Frequency of white colonies is shown highlighted in red underneath each image.

To confirm the spontaneous suppressors identified, three of the mutations were separately introduced into a Δ*radD*Δ*recG* strain (adding the suppressor mutation prior to the introduction of one of the two helicase deletion mutations; with Δ*radD* usually added last). The PriA S278A, PriA A520P and *P_recA_* mutations were all able to eliminate the growth defect, completely abrogating the extended lag phase of a Δ*radD*Δ*recG* strain (Figure 5A). This demonstrates that the identified mutations are responsible for the observed suppression. Each suppressor mutant was also tested for complementation by monitoring the appearance of white colonies when the pRC7-*recG* plasmid was introduced into the triple mutant strains. Strains in which the Δ*radD*Δ*recG* was suppressed by PriA S278A, PriA A520P or *P_recA_*, lost the plasmid (indicating no requirement to retain RecG function) at a frequency equal to or greater than wild type strains (Figure 5B, C). Total CFU are reported after 24 h. Counts were taken at 16 and 24 h. White versus blue colony numbers stayed the same across both time points. Each of these three mutations, on their own, thus reproduce the suppression effect in its entirety.

The appearance of a suppressor in the *recA* promoter suggested to us that additional avenues of suppression of the Δ*radD*Δ*recG* growth defect might exist. We reasoned that the concentration of suppressors in the *priA* gene might simply reflect a multitude of SNP mutational paths to suitable functional *priA* suppressors, while alternative suppressors might require a more unlikely mutational change or complete inactivation of a gene or genes. To explore this idea, we abandoned the screen of spontaneous and random suppressor generation and tried a more directed approach. We made a series of triple mutants in which a candidate gene was deleted and combined with Δ*radD*Δ*recG* (in each case adding the *radD* deletion last) in a *lac^−^* background and tested them for suppression. Triple mutants combining deletions of the *rep*, *ruvB*, or *rarA* genes with Δ*radD*Δ*recG* failed to elicit suppression, with the strains very difficult to construct or maintain. However, good suppression was obtained when *recF*, *recO*, or *uup* deletions were introduced, as described in the next two sections.

### Deleting *recO* or *recF* suppresses the Δ*radD*Δ*recG* growth defect

**Figure 6.**
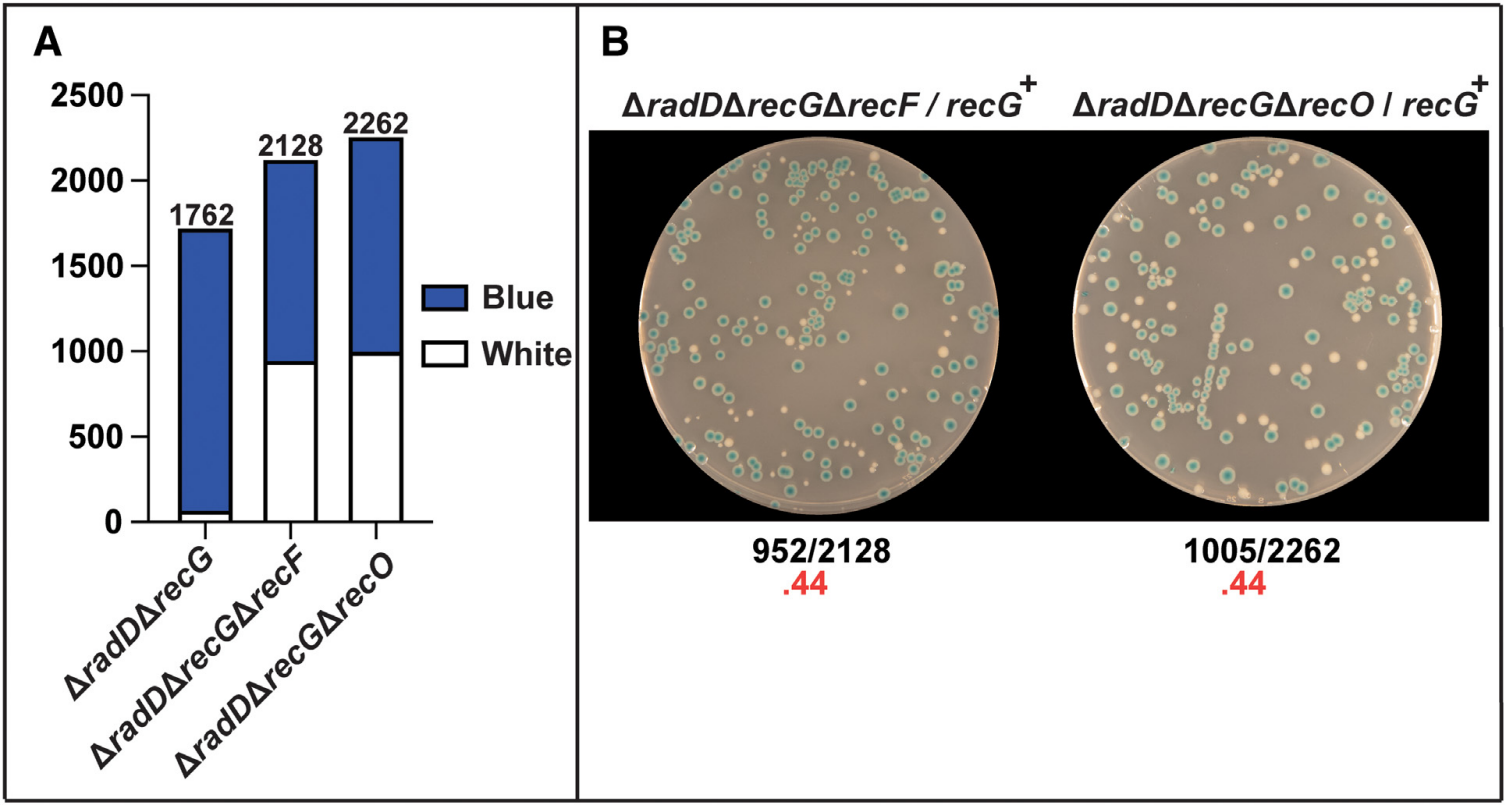
(**A**) Stacked bar graph quantifying results of pRC7 assay of Δ*radD*Δ*recG* with either *recF* or *recO* deleted. (**B**) Images of plates after 24 h of results for Δ*radD*Δ*recG* with *recF* or *recO* deleted.

If reducing the concentration of RecA in the cell could suppress (the *P*_recA_ mutation), we wondered if blocking RecA loading could also suppress. The RecFOR proteins are implicated in loading RecA protein primarily in postreplication gaps (13,45–49). We made the triple Δ*radD*Δ*recG*Δ*recO* and Δ*radD*Δ*recG*Δ*recF* strains and tested them for suppression. Both strains were able to rescue the Δ*radD*Δ*recG* growth defect and to restore the appearance of white colonies to a ratio of 0.44 when the pRC7-*recG* plasmid was introduced (Figure 6A, B). This supports the idea that the deleterious Δ*radD*Δ*recG* phenotype involves an inability to resolve recombination intermediates being generated at a stalled replication fork or postreplication gap.

### A full gene deletion of Uup suppresses the Δ*radD*Δ*recG* growth defect

Uup is a UvrA-like Class II ABC system that binds Holliday junctions. RadD and Uup help define at least two pathways for resolution of branched DNA intermediates during template switching in post replication gaps (33). Uup and RadD are responsible for the stabilization of tandem repeats that are susceptible to deletion (33). These deletion events mimic the RecA-mediated gap repair pathway. However, they are RecA-independent and can be mutagenic. Due to the ubiquitous nature of Holliday junctions in other pathways, we hypothesized that RadD and Uup may be involved in other repair processes. And as RecG and RadD appear to complement each other, we wondered if Uup and RecG might be involved in the same pathway. We thus decided to test if deleting *uup* suppresses the defect seen in the Δ*radD*Δ*recG* mutant strain.

**Figure 7.**
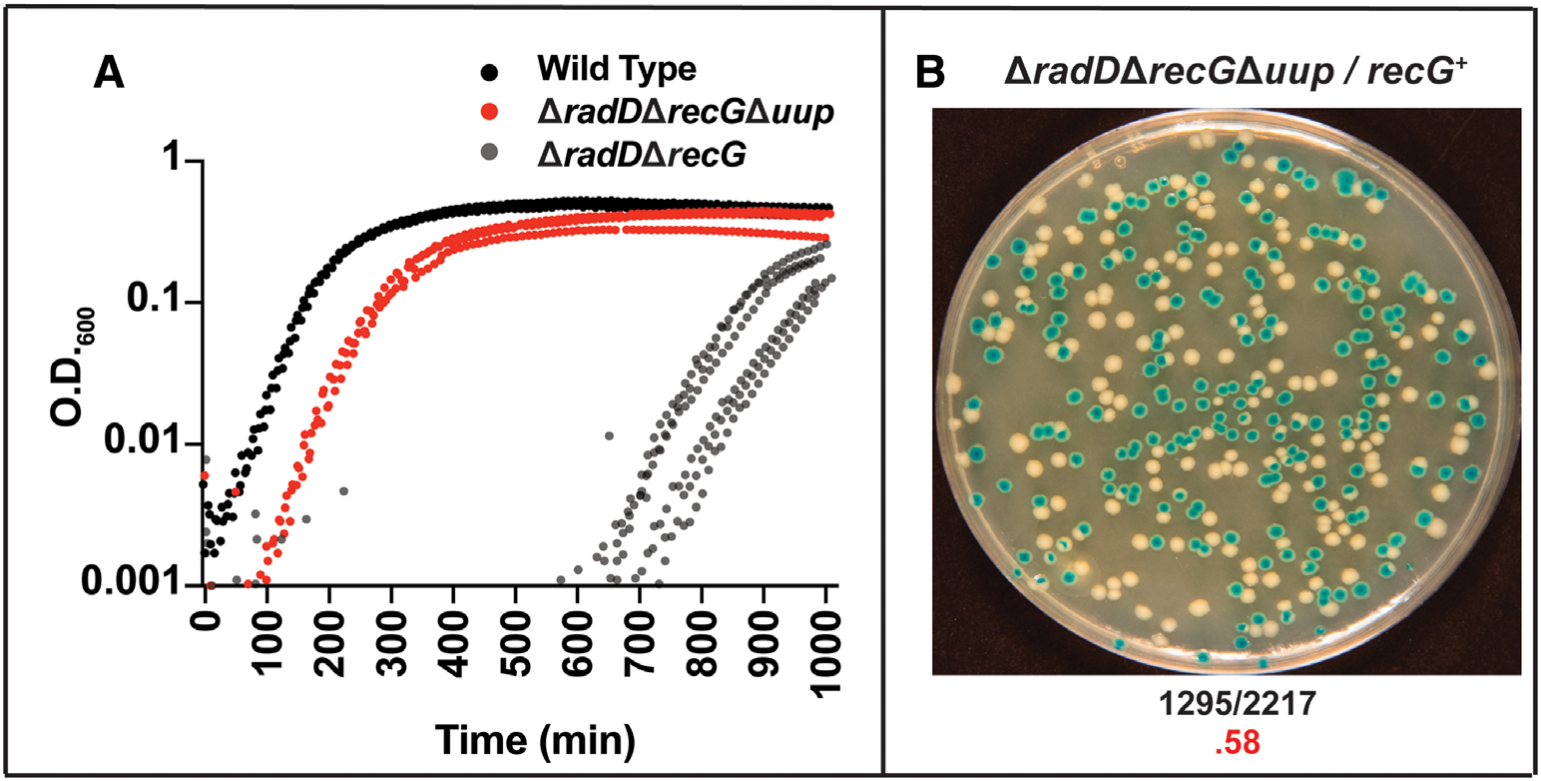
(**A**) A minimum of 3 Log scale OD_600_ versus time traces of Δ*radD*Δ*recG*Δ*uup* (red) compared to wild type (black) and Δ*radD*Δ*recG* (gray). (**B**) Images of plates after 24 h of pRC7 results of Δ*radD*Δ*recG*Δ*uup* strain with frequency of white colony formation highlighted below in red.

As seen with the suppressors already described, deleting *uup* rescued colony size and suppressed the growth defect of Δ*radD*Δ*recG* (Figure 7). The triple Δ*radD*Δ*recG*Δ*uup* mutant did produce a growth lag, similar to that observed in the Δ*recG* single mutant. Combining Δ*uup* with Δ*radD*Δ*recG* was also examined in mini-F plasmid assay. Here, loss of Uup function restored the appearance of white colonies to levels comparable to that seen for wild type strains. Thus, a deletion of *uup* appears to be effective in suppressing the growth defect of the Δ*radD*Δ*recG* double deletion mutant strain.

### Effects of deleting combinations of *uup*, *radD*, and *recG* on sensitivity to DNA damaging agents

**Figure 8.**
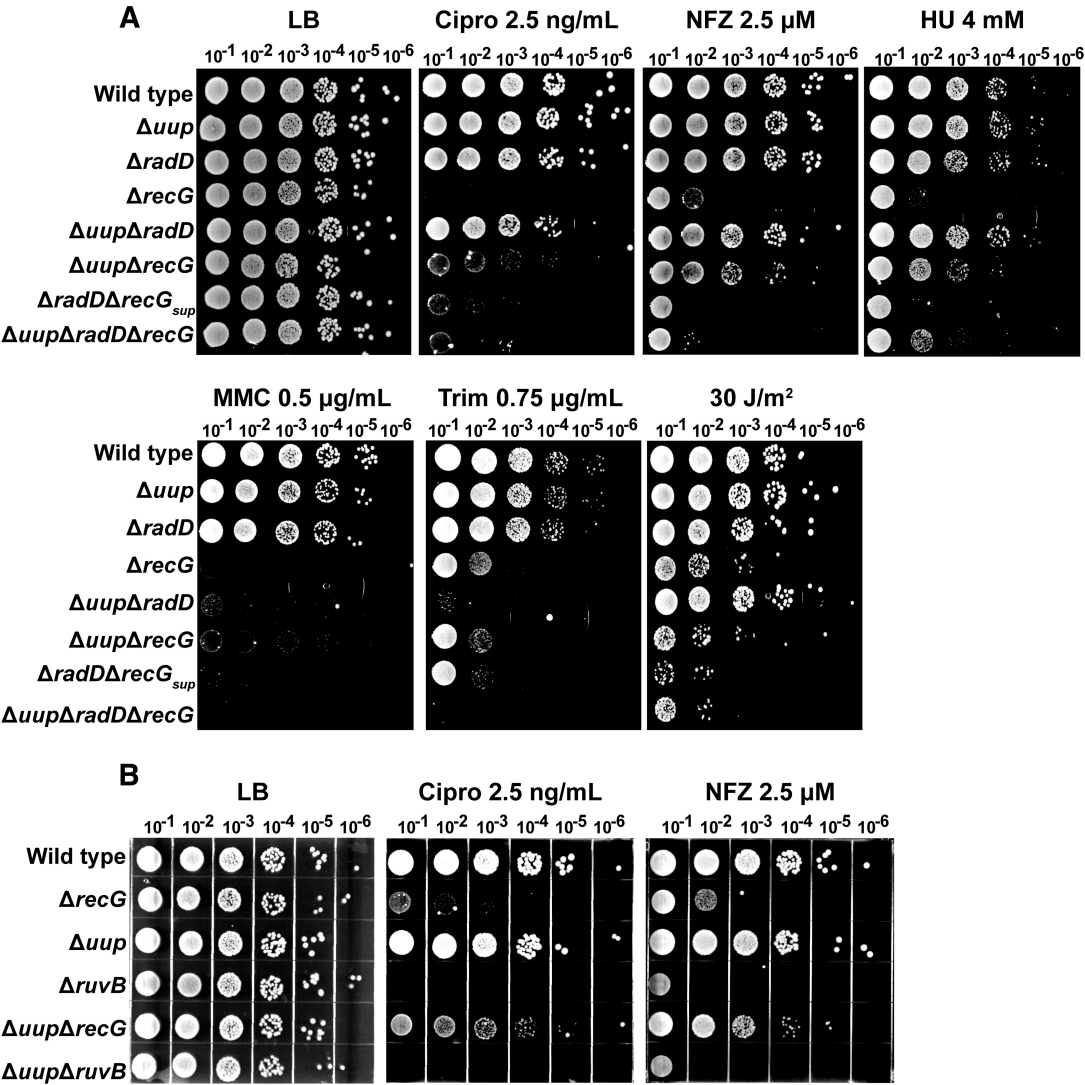
(**A**) Sensitivity assays with all possible *radD, recG* and *uup* deletion combinations. Spot plates indicate compound, dose, and dilution above each plate. LB, Cipro, NFZ, HU, MMC, Trim and UV are Luria Broth, ciprofloxacin, nitrofurazone, hydroxyurea, mitomycin C, trimethoprim and ultraviolet light, respectively. The Δ*radD*Δ*recG* strain used in Figure 8 has undergone an extra overnight growth period so that its treatment is consistent with that of the other strains. Its facile growth on some of these plates demonstrates that it has picked up a suppressor. It is included for the sake of completion but is designated Δ*radD*Δ*recG*_supp_ to highlight this status. (**B**) Sensitivity assays with *uup recG* and *ruvB* gene deletion combinations. Damaging agent, dose and dilution are listed above each plate.

We wished to explore the potential connection between *uup* and *recG* further, determining whether the effects of suppression by deleting *uup* could be extended to conditions of stress. We treated all possible *radD*, *recG* and *uup* gene deletion combinations with various DNA damaging agents (Figure 8). The dose used was tailored to the high sensitivity of the Δ*recG* and Δ*radD*Δ*recG* strains to DNA damaging agents. Multiple survival patterns were observed, varying not only with the mutants employed but also with the different DNA damaging agents. The latter effects presumably reflect variations in the pathways with which particular types of DNA lesions are normally resolved.

#### Pattern 1

Loss of Uup or RadD function alone had no significant effects on their own with any DNA damaging agent (Figure 8A). Loss of Uup and RadD together also had minimal effects except in the cases of Mitomycin C (MMC) and Trimethoprim (Trim) (increased sensitivity of the double deletion mutant has also been noted for Ciprofloxacin (Cipro) at levels higher than used here (33)). These results are consistent with earlier observations and provide one rationale for why Uup and RadD were largely overlooked until recently.

#### Pattern 2

Deletion of *uup* in combination with *recG* strongly suppressed the high sensitivity of Δ*recG* strains to Cipro, Nitrofurazone (NFZ), Hydroxyurea (HU), and MMC. This effect is not seen for either Trimethoprim or UV irradiation. Survival on MMC wasn’t as greatly enhanced as the other three agents. At higher doses of NFZ and Cipro, the Δ*uup*Δ*recG* began to exhibit some sensitivity when compared to wild type (Supplemental Figure S2). This result in general suggests that many of the deleterious effects of a *recG* deletion (but not all) are dependent on the presence of a functional Uup protein. In the Discussion, we offer a hypothesis for a functional relationship between Uup and RecG that can explain these observations. The result also indicates that RadD can make a substantial contribution to survival when both RecG and Uup are missing.

#### Pattern 3

The addition of a *radD* deletion to construct the Δ*radD*Δ*recG*Δ*uup* triple mutant generally eliminates the suppressive effect of a *uup* deletion on the DNA damage sensitivity of a Δ*recG* strain. In some cases (Trim, MMC), the sensitivity of the triple mutant is somewhat greater than that seen with Δ*recG* alone. This result again speaks to the existence of multiple, partially redundant pathways for repair, with a key alternative path blocked when *radD* is eliminated. Thus, although growth rates are restored under normal conditions with the triple mutant, it remains highly sensitive to elevated levels of DNA damage.

#### Pattern 4 (Figure 8B)

The suppression that a *uup* deletion confers on a Δ*recG* phenotype does not extend to *ruvB*. RuvB is part of the resolvasome that is responsible for the resolution of HJs and replication fork processing (50,51). RuvB is also involved in replication fork reversal (3,52–54). There are no conditions in our trials where Δ*uup* increases the survival of a Δ*ruvB* strain, and one condition (MMC) where the sensitivity to DNA damage is exacerbated. The suppressive effects of a *uup* deletion are thus specific to *recG*.

We note that the Δ*radD*Δ*recG* strain used in Figure 8 has undergone an extra overnight growth period so that its treatment is consistent with that of the other strains. Its facile growth on some of these plates demonstrates that it has picked up a suppressor. It is included for the sake of completion but is designated Δ*radD*Δ*recG*_supp_ to highlight this status.

The various DNA-damaging agents utilized in Figure 8 function in different ways to inflict damage and affect replisome progress. Ciprofloxacin is a quinolone that inhibits DNA Gyrase. Inhibition of DNA Gyrase leads to a replication roadblock. Replisome stalling occurs ∼10 bases upstream of the halted gyrase cleavage site (55). Nitrofurazone at low doses induces base lesions in the form of N^2^-alkyl deoxyguanosine that relies on the nucleotide excision repair machinery to repair (56,57). Hydroxyurea is an inhibitor of ribonucleotide reductase and will deplete the nucleotide pool leading to replication stalling and disassociation (58). All three of these compounds have the potential to trigger formation of a reversed fork intermediate. Mitomycin C creates protein and DNA crosslinks that can pose stalling risks to the replisome machinery. Trimethoprim triggers rapid thymine depletion which then cascades to further DNA damage (59).

### Uup suppresses the growth defect but not cell filamentation in the Δ*radD*Δ*recG* strain

**Figure 9.**
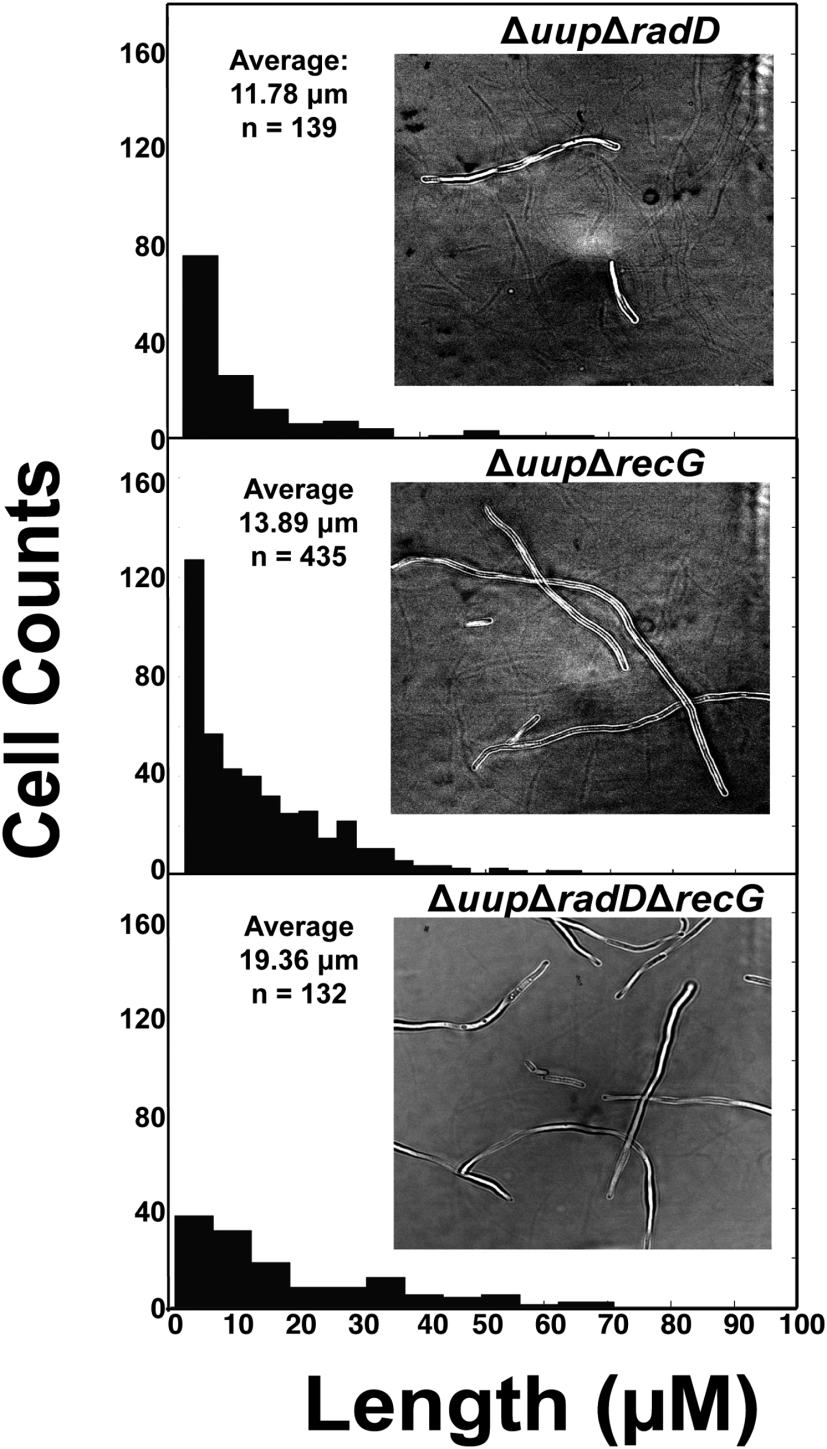
Cell filamentation measurements of Δ*uup*Δ*radD*, Δ*uup*Δ*recG* and Δ*uup*Δ*radD*Δ*recG* strains. Average length, number of cells and a bright field image are displayed for each strain.

We also wished to determine the status of the cells when the Δ*radD*Δ*recG* strain is suppressed by Δ*uup*. We had previously observed that strains lacking Uup function filament rather extensively under normal growth conditions. As seen in Figure 9, strains lacking RecG did not alleviate the filamentation, but rather exacerbated it. The Δ*radD*Δ*recG*Δ*uup* cells filamented extensively with the average cell length of these cells exceeding 19 μm. Thus, even if the growth defect of cells lacking RadD and RecG is suppressed by deleting Uup, deficiencies in replication, repair, and cell division are still abundantly evident.

### Uup is required for SDR-dependent growth in *rnhA dnaAts* mutants

To further investigate the relationship between Uup and RecG, we explored a process with which RecG is closely associated, stable DNA replication or SDR. SDR is origin-independent replication, initiating at readily detectable levels in cells lacking the function of RnaseH or RecG (60–62). In *E. coli rnhA* mutants, SDR supports cell growth in the absence of *oriC* function, presumably via replication initiation at unprocessed R-loops scattered about the genome (61). In *E. coli recG* mutants, SDR is largely restricted to the terminus region where over-replication is initiated when RecG is unable to resolve structures created by fork collisions (62). Cell growth in the absence of *oriC* is not supported in a *ΔrecG* strain unless additional mutations in *tus* (to allow forks to escape the terminus region) and *rpoB* (to relieve replication-transcription conflicts) are also introduced (62). An *rnhA recG* double mutant is inviable and cannot be constructed, (63) presumably because RecG is needed to process the fork collisions that occur when *oriC*-independent replication is initiated in the absence of RnaseH. We reasoned that if Uup acted upstream of RecG, an absence of Uup function might also affect growth in a strain lacking RnaseH when *oriC* function was compromised.

**Figure 10.**
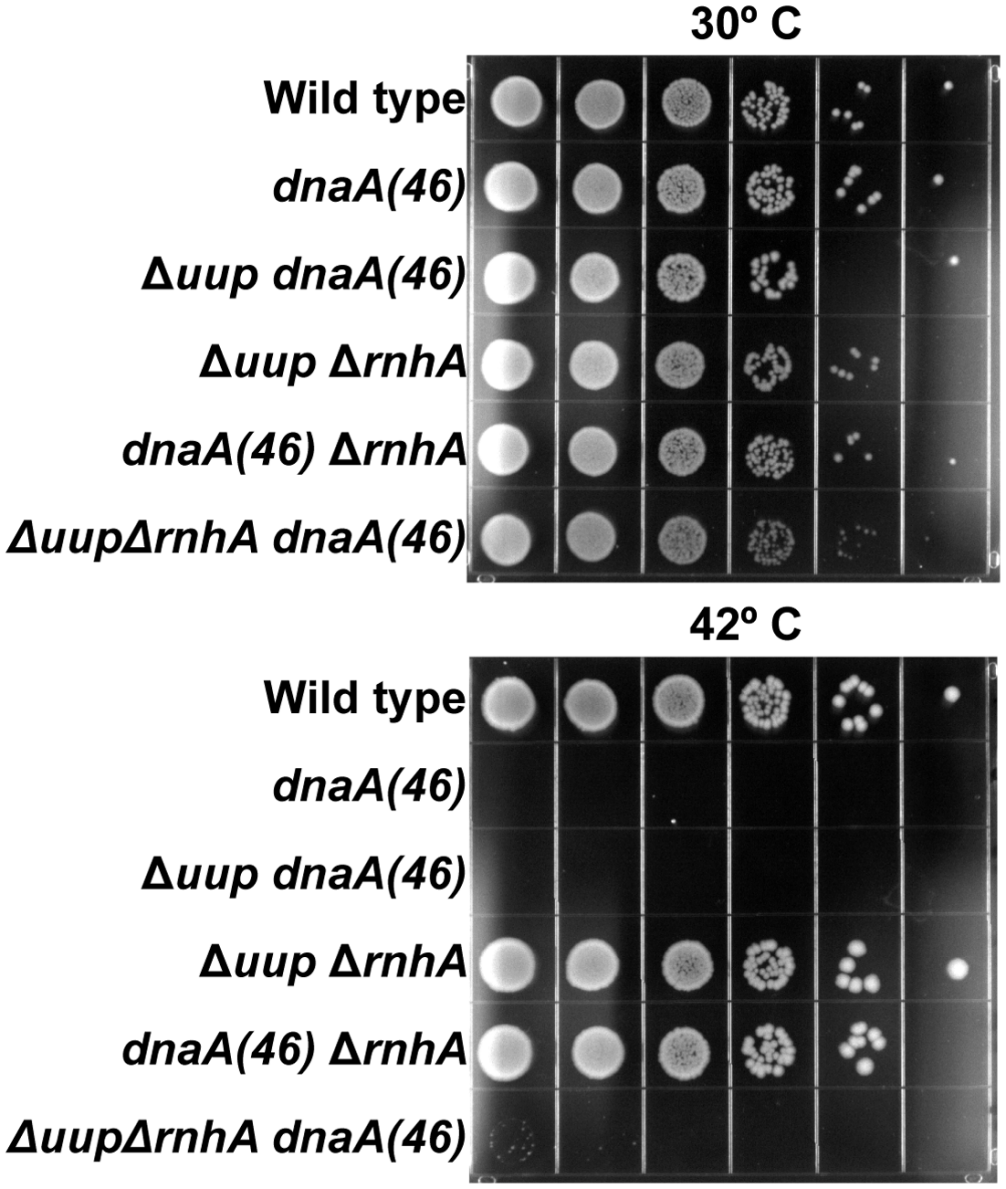
Spot plates grown at permissive (30°C) or restrictive (42°C) temperature. The *dnaA(46)* allele cannot grow at 42°C unless accompanied by *rnhA* deletion shown in the first and fourth rows of both plates. Adding a *uup* deletion to *dnaA(46)*Δ*rnhA* strain restores temperature sensitive growth as shown on the last row of both plates.

Results are presented in Figure 10. We began with a *dnaA(46)* mutation which supports normal *oriC*-dependent replication at 30°C but not at 42°C (64). To this we added a *ΔrnhA* mutation, a *Δuup* mutation, or both. We also tested a *ΔrnhAΔuup* double mutant without the *dnaA* mutation. At 30°C, the WT and all of the mutant combinations grow similarly. At 42°C, the WT and *dnaA(46)ΔrnhA* double mutant grew as expected. The *ΔrnhAΔuup* double mutant, unencumbered with a temperature sensitive DnaA protein, also grew. The *dnaA(46)* single mutant does not grow, again as expected. The *Δuup dnaA(46)* control double mutant did not grow. Most important, when *Δuup* was added to *dnaA(46)ΔrnhA*, the growth observed in the *dnaA(46)ΔrnhA* double mutant was entirely eliminated. As growth in this mutant is dependent on RecG function, the result provides another possible connection between Uup and RecG.

### Mutations in *priA* and *P_recA_* suppress Δ*radD*Δ*recG* defect by mitigating Δ*recG* effects but still exhibit high SOS induction

**Figure 11.**
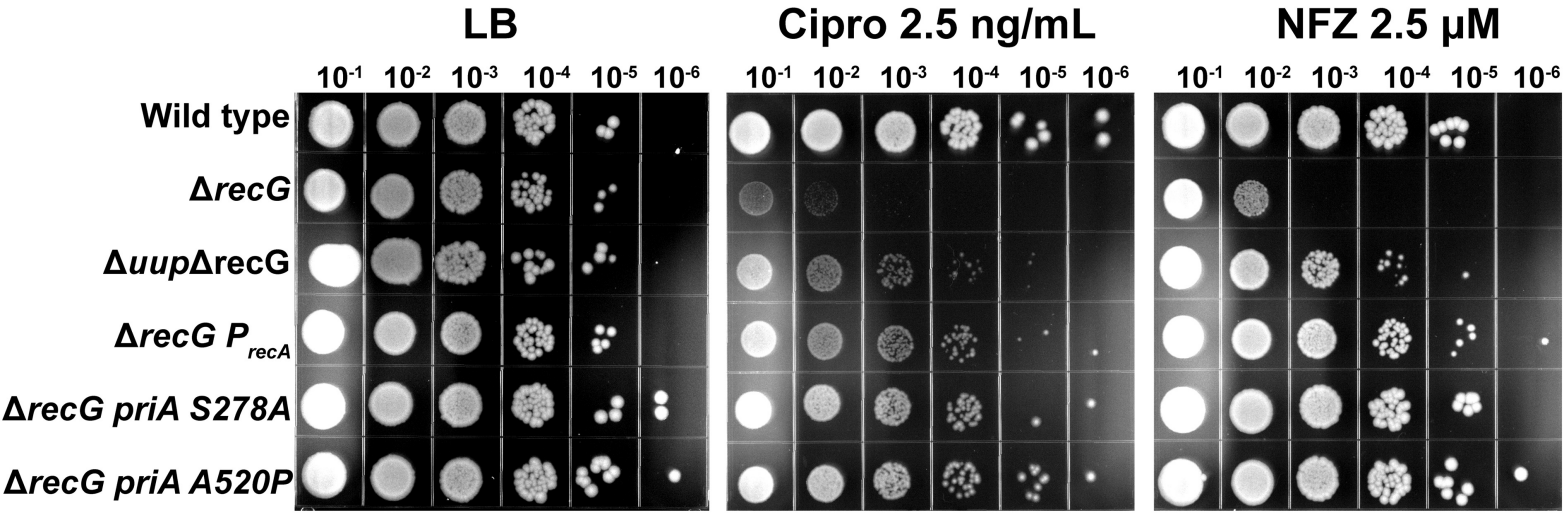
Sensitivity assays exploring Δ*recG* cells with either *uup, priA S278A, priA A520P* or *P_recA_* mutation added. Compound, dose and dilution are labeled at the top of each plate. LB, cipro and NFZ are Luria Broth, ciprofloxacin and nitrofurazone, respectively.

The suppression of *recG* sensitivity to damaging agents by a *uup* deletion made us question if all accumulated suppressors are directed at alleviating the consequences of deleting *recG*. The double mutants of Δ*recG priA* S278A, Δ*recG priA A520P*, and Δ*recG P_recA_*were made and treated with cipro or NFZ (Figure 11). All three suppressors were able to rescue survival of the *recG* mutant. Figure 2 shows that despite the presence of a suppressor as a result of extended growth prior to the experiment (see discussion of Figure 2), a Δ*radD*Δ*recG* strain still shows high SOS induction. We wanted to determine if SOS induction again occurred when a defined suppressor was present, by incorporating both *priA* and *PrecA* suppressor mutations into a Δ*radD* strain before deleting *recG*. We found that both *priA A520P* and the *P_recA_* mutation (in the Δ*radD*Δ*recG* background) exhibited increased levels of SOS in the absence of stress. The *priA S278A* mutant, however, does not (Supplemental Figure S1). The results suggest that the main effect of the suppressors is to abrogate the deleterious effects of the *recG* deletion. The results also indicate that the *priA* suppressor mutations are not all equivalent in their effects on PriA activity.

## DISCUSSION

This work leads to two major conclusions with several subsidiary observations. One major result is that loss of both RadD and RecG function generates a severe growth defect in *E. coli* under normal growth conditions in rich media but otherwise in the absence of stress. At least some cells survive to generate suppressor mutations. This indicates that replisome challenges requiring either RecG or RadD intervention are a feature of virtually every replication cycle. The second conclusion is that RadD and Uup are both important functions in the repair processes involving replication fork stalling and the processing of postreplication gaps. RadD is essential to growth in the absence of RecG. Uup appears to function in a pathway or pathways that also feature RecG, likely acting upstream of RecG in at least some key situations.

Subsidiary observations include the following: (i) RadD and RecG function in distinct pathways that both contribute to maintenance of genomic integrity during replication. The work highlights the importance of RadD in at least one of those pathways. (ii) Proteins that create structures or situations requiring the action of RadD or RecG include (but are probably not limited to) RecA, RecO, RecF, Uup and PriA. (iii) Suppression of the *ΔrecGΔradD* phenotype relieves the barrier to growth. However, the cells remain very sensitive to DNA damaging agents. The RadD and RecG proteins play an important role in DNA repair that cannot be completely bypassed by alternative pathways. (iv) There appears to be some set of lesions or replication barriers for which pathways involving RecG or RadD are the primary paths to repair. At least for these events, RadD and RecG are among the first responders. Translesion (TLS) DNA synthesis repair pathways are still intact, but they are unable to overcome the damage that persists in a Δ*radD*Δ*recG* strain.

**Figure 12.**
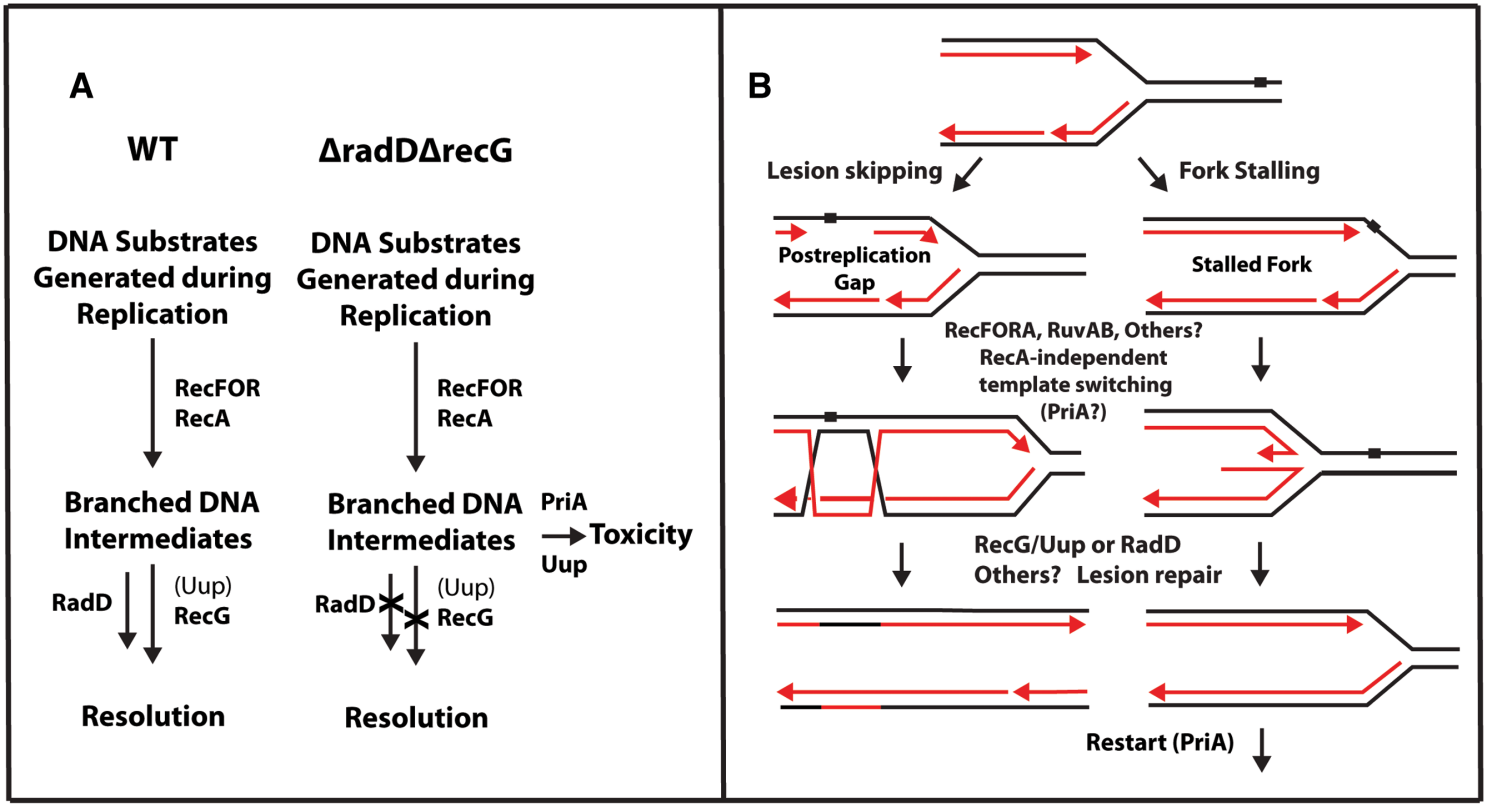
(**A**) Functional scheme showing how each gene fits into the various stages of intermediate processing. In the absence of RecG or RadD, a buildup of intermediates leads to toxic processing that is dependent on Uup and PriA. (**B**) Schematic demonstrating repair activities facilitated by RecG or RadD in a postreplication gap and stalled replication fork. Either protein may be capable of branch migrating Holliday Junctions formed in postreplication gaps or to revert a regressed fork into a substrate suitable for replication restart.

The identification of suppressors allows us to outline likely paths for DNA intermediate processing (Figure 12A). The initial DNA substrate generated during replication or as a result of replisome stalling is processed by the RecA recombinase via the RecFOR pathway. With all proteins present, the RecG or RadD-dependent pathways facilitate productive repair and resolution. In the absence of both proteins, persistent DNA intermediates will become targets for deleterious processing due to the perturbation of normal repair flow. The types of branched DNA intermediates likely to be targets for these resolution pathways, or at least some of them, are shown in Figure 12B.

The general view of two repair pathways, one with RecG (sometimes in partnership with Uup) and the other with RadD, is based not only on the growth defect and suppression patterns, but also on the DNA damage sensitivity patterns and observed effects on SDR. Many of the deleterious effects of a *recG* deletion depend upon the continued presence of Uup. The DNA damage sensitivity to Cipro, NFZ, HU and MMC exhibited in a *ΔrecG* strain is greatly ameliorated if *uup* is also deleted. In addition, the SDR that supports *oriC*-independent growth in a strain lacking RnaseH is suppressed if Uup is missing. While not constituting final proof, all of these observations lead to an obvious hypothesis: that Uup functions upstream of RecG.

Even if our hypothesis that Uup functions upstream of RecG is correct in some contexts, Uup is not required in all situations in which RecG contributes. We cite four examples of data indicating that Uup is not needed for RecG function in all contexts: (a) In no case are the effects of a *uup* deletion as phenotypically deleterious as a *recG* deletion. (b) A lack of Uup eliminates growth in a *dnaA(46)ΔrnhA* double mutant at nonpermissive temperatures. A *ΔrnhAΔrecG* double mutant cannot be constructed, with no viability at any temperature with or without a *dnaA(46)* mutation. (c) Whereas a *ΔrecGΔradD* strain cannot grow, a *ΔuupΔradD* strain grows well in the absence of stress (33). (d) Eliminating Uup does not affect the sensitivity of a *recG* deletion to UV irradiation or Trimethoprim although it does suppress the *ΔrecG* sensitivity to a number of other agents. Overall, the results suggest an association of Uup with RecG that is limited to particular situations or substrates.

The very strong growth defect in a Δ*radD*Δ*recG* strain, coupled to the reliable generation of numerous suppressor mutations, provides a powerful experimental *entre* into the workings of the underlying repair pathways. The subsidiary observations come largely from the identity of the suppressor mutations. Spontaneous suppressors identified to date compromise the function of PriA (many) or arise in the *recA* promoter so as to lower RecA expression (one). It is unlikely that we have saturated the possibilities for suppression. The concentration of suppressors in the *priA* gene may simply reflect a multitude of mutational paths to suitable functional *priA* suppressors. Many single nucleotide polymorphisms in the *priA* gene appear to alter PriA function in a suitable manner. Facile success in *priA* can have the consequence of obscuring other avenues to suppression. Alternatives might require a more unlikely mutational change or complete inactivation of a gene or genes. By exploring a few logical possibilities, we have found additional suppressors that affect RecA loading onto SSB-coated ssDNA (elimination of RecO or RecF) or eliminate Uup function. These suppressors do not immediately suggest a common mechanistic origin. Reduction in RecA-mediated fork reversal at a stalled replisome, or RecA-mediated strand exchange in a postreplication gap, may reduce the numbers of branched intermediates requiring intervention by the RecG or RadD helicase functions. Rescue of the Δ*radD*Δ*recG* strain's viability by eliminating the RecA-loading functions RecO or RecF supports this idea. In the absence of RecG or RadD function to restore reversed forks or resolve RecA intermediates in post replication gaps, PriA may engage in toxic activity (28). PriA has figured prominently in the suppression of *recG* phenotypes in earlier studies (15,20,21,31,62,64).

The *uup* suppression is more difficult to explain mechanistically but may arise from the putative functional relationship between Uup and RecG. Based entirely on its structural relationship to UvrA and its documented binding to Holliday junctions *in vitro*, we have hypothesized that Uup is a DNA scanner that binds to Holliday junctions. Thus bound, Uup may recruit other repair functions to deal with the bound DNA species, with RecG now a prime candidate for recruitment. Based on the positive effects of a *uup* deletion on the DNA damage sensitivity of a strain missing RecG function, a plausible (but doubtless not unique) scenario can be put forward as a working hypothesis. Uup scans DNA for Holliday junctions and binds to them. RecG is recruited, and then migrates the branch to either restore a fork structure or resolve an intermediate in postreplication gap repair. If RecG is missing, Uup may bind to the Holliday junction in such a way as to block or constrain other potential paths of resolution. If Uup is also missing, the deleterious effect of RecG loss is ameliorated as other paths take over. RadD represents an important component of the major alternative path.

The work further defines the function of the enigmatic helicase RadD. Like RecG, RadD appears to be involved in many repair processes. Deletion of both helicases results in a nearly inviable strain unless accompanied by suppressor mutations. The requirement for both proteins can be explained by a few different mechanisms. (i) RadD has complementary activity to RecG. RadD can bind fork structures, suppress crossover products, and has an interaction with the replisome hub protein SSB (33,35). These observations, while seemingly disparate, become logical when combined with the severe growth defect of the Δ*radD*Δ*recG* strain. RadD can supplement for lost RecG function at an abandoned fork or resolve D-loops formed in gaps by RecA to prevent SDR initiation. (ii) RadD can be viewed as a first responder to replisome roadblocks. This idea establishes RadD as a ‘housekeeping’ helicase localized to the replisome or gaps through its SSB interaction.

The dependence of cells on either RadD or RecG present exciting new avenues of study. RadD can possibly provide insights on how specific lesions dictate repair pathways. Uup may be a modulator of RecG. Further investigation of these ideas is currently underway.

## Supplementary Material

gkaa579_Supplemental_File
