# Supplementary material for: Resolving Toxic DNA repair intermediates in every E. coli replication cycle: critical roles for RecG, Uup and RadD
Source: Nucleic Acids Res. 2020 Jul 9;48(15):8445–60. doi: 10.1093/nar/gkaa579 (PMC7470958; doi:10.1093/nar/gkaa579)
Supplement: gkaa579_Supplemental_File [file gkaa579_supplemental_file.pdf]

Supplementary Material for Romero et al.

Resolving Toxic DNA Repair Intermediates in every *E. coli* Replication Cycle: Critical Roles for RecG, Uup, and RadD

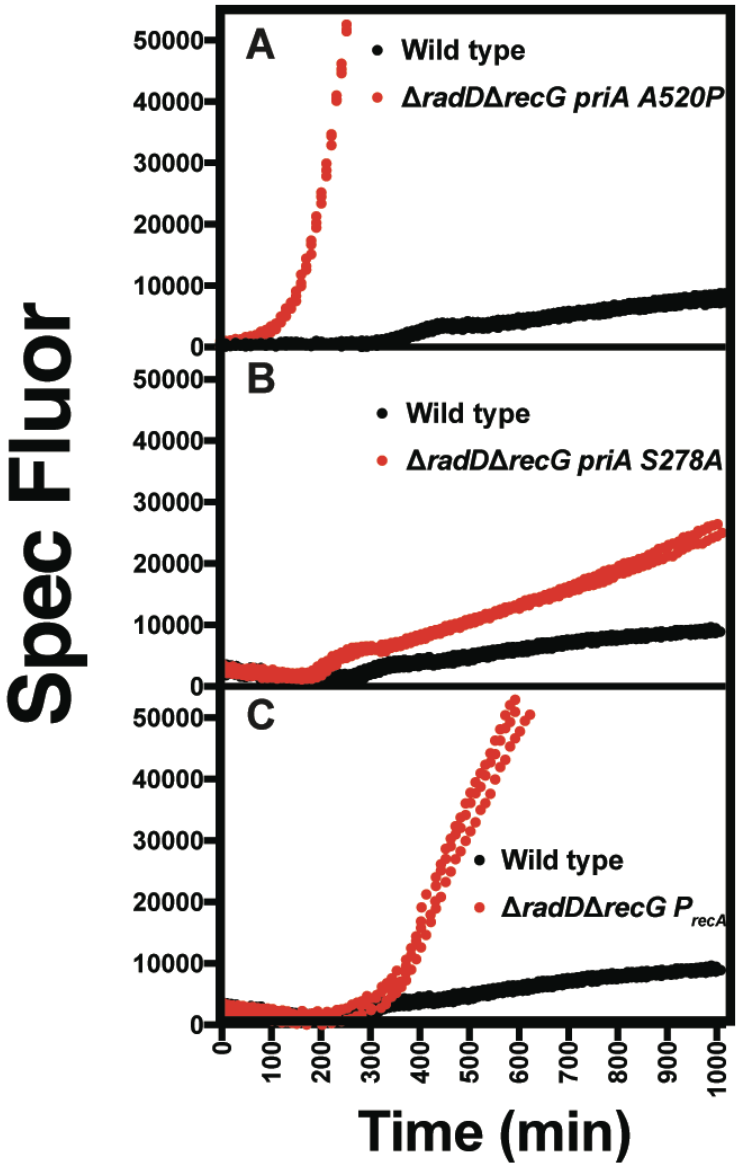

Figure S1. Fluorescent reading for SOS induction in ΔradDΔrecG background with each suppressor mutation over time. Wild type trace is in black and red traces have the suppressor mutation added.

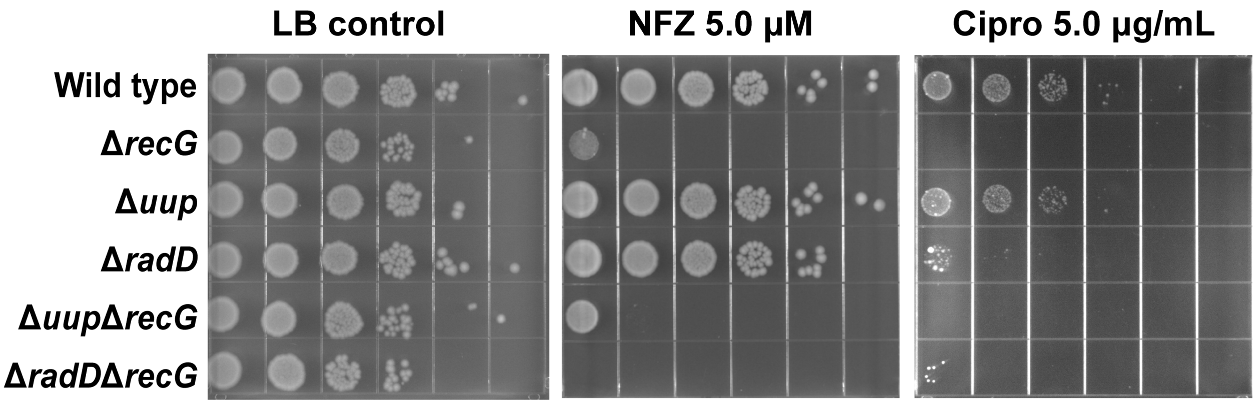

Figure S2. Spot plates showing restored sensitivity to damaging agents at higher doses of nitrofurazone and cipro for ΔuupΔrecG strain.

Ref ID	Ref Pos	Gene Name	Base Change	Aminco Acid Change
NC_000913.3	132923	acnB	C>T	p.(=)
NC_000913.3	803662	ybhJ	C>A	p.L54I
NC_000913.3	1170381		T>C	
NC_000913.3	1196220	icd	C>T	p.(=)
NC_000913.3	1196232	icd	C>T	p.(=)
NC_000913.3	1196245	icd	T>C	p.(=)
NC_000913.3	1196247	icd	A>G	p.(=)
NC_000913.3	1196277	icd	C>T	p.(=)
NC_000913.3	1196280	icd	G>C	p.(=)
NC_000913.3	1196283	icd	A>G	p.(=)
NC_000913.3	1196292	icd	C>T	p.(=)
NC_000913.3	1196304	icd	G>A	p.(=)
NC_000913.3	1196316	icd	T>A	p.D398E
NC_000913.3	1196325	icd	A>G	p.(=)
NC_000913.3	1196352	icd	C>A	p.D410E
NC_000913.3	1351174		G>A	
NC_000913.3	1523360	yncE	T>C	p.(=)
NC_000913.3	1905761	mntP	G>A	p.G25D
NC_000913.3	2173361	gatC (pseudo)	CC > del 2	
NC_000913.3	2823830		A>G	
NC_000913.3	3560455	glpR (pseudo)	- > ins G	
NC_000913.3	3938501	rbsR	T>G	p.L92R
NC_000913.3	4124129	cytR	G>A	p.Q110.
NC_000913.3	4296380		- > ins CG	

Table S1. Sequencing data for the ΔradDΔrecG isolate harboring the PrecA mutation. The suppressor mutation is highlighted in yellow.
